# Linear Mixed-Effects Models to Describe Individual Tree Crown Width for China-Fir in Fujian Province, Southeast China

**DOI:** 10.1371/journal.pone.0122257

**Published:** 2015-04-15

**Authors:** Xu Hao, Sun Yujun, Wang Xinjie, Wang Jin, Fu Yao

**Affiliations:** Key Laboratory for Silviculture and Conservation of Ministry of Education, College of Forestry, Beijing Forestry University, Beijing, PR China; Pennsylvania State University, UNITED STATES

## Abstract

A multiple linear model was developed for individual tree crown width of *Cunninghamia lanceolata* (Lamb.) Hook in Fujian province, southeast China. Data were obtained from 55 sample plots of pure China-fir plantation stands. An Ordinary Linear Least Squares (*OLS*) regression was used to establish the crown width model. To adjust for correlations between observations from the same sample plots, we developed one level linear mixed-effects (*LME*) models based on the multiple linear model, which take into account the random effects of plots. The best random effects combinations for the *LME* models were determined by the Akaike’s information criterion, the Bayesian information criterion and the -2logarithm likelihood. Heteroscedasticity was reduced by three residual variance functions: the power function, the exponential function and the constant plus power function. The spatial correlation was modeled by three correlation structures: the first-order autoregressive structure [AR(1)], a combination of first-order autoregressive and moving average structures [ARMA(1,1)], and the compound symmetry structure (CS). Then, the *LME* model was compared to the multiple linear model using the absolute mean residual (*AMR*), the root mean square error (*RMSE*), and the adjusted coefficient of determination (adj-*R* ^2^). For individual tree crown width models, the one level *LME* model showed the best performance. An independent dataset was used to test the performance of the models and to demonstrate the advantage of calibrating *LME* models.

## Introduction

China-fir (*Cunninghamia lanceolata* (Lamb.) Hook) is the most commonly grown afforestation species in southeast China because of its fast growth and good wood qualities. It is widely used for buildings, furniture, bridge construction and many other purposes. According to the National Continuous Forest Inventory, approximately 11.26 million hectares and 734.09 million cubic meters of China-fir were distributed over 10 provinces in China in 2010.

Growth and yield models are commonly used for forest management planning because they can simulate stand development and production under various management alternatives [1; 2]. As an important tree variable, the crown width (CW) of individual trees is a fundamental component of forest growth and yield prediction frameworks [3; 4], and it is also crucial for assessing the competitive level, tree vigor, microclimate, biological diversity, mechanical stability, fire susceptibility and behavior under wind stress, amongst other features [5]. The tree crown displays the leaves to capture radiant energy for photosynthesis and is strongly correlated with tree growth [6]. Therefore, measurements of the tree crown are often made to aid the understanding and quantification tree growth [7]. However, it is excessively costly and time consuming to measure the crown width of trees [8; 9]. As a result, it is necessary to establish accurate crown width models for forest managers to predict crown width precisely based on the crown data from adequate numbers of sample trees within different sample plots.

Regression analysis, such as the Ordinary Linear least Squares (*OLS*) regression, is the most commonly used statistical method in forest modeling [10]. Most crown width models are simple linear or nonlinear functions of diameter at breast height (DBH), estimated using linear or nonlinear regression [8; 9]. The fitting data for crown width models are usually collected by measurements of trees within different plots, also known as cross-sectional data [11; 12]. The hierarchical nature of the data results in spatial correlation among measurements made in the same sampling unit (i.e., plot) [13]. However, the hierarchical structure is often ignored and independence of observations is assumed [8–10; 14; 15]. Furthermore, the data are autocorrelated and cannot be considered independent samples of the basic plot population [13]. The *OLS* regression assumption of independent residuals is therefore violated, biasing the estimates of the standard error of the parameter estimates [16].

Linear mixed-effects (*LME*) models that include both fixed-effects and random-effects provide an efficient means of analyzing some kinds of cross-sectional data [17; 18]. The fixed-effects parameters are associated with an entire population or with certain repeatable levels of experimental factors, and the random-effects parameters are related to individual experimental units drawn at random from a population. These parameters account for spatial correlation by defining the covariance structure of the model’s random component and by using this structure during parameter estimation. Because of their advantages, *LME* models provide an efficient statistical method for explicitly modeling hierarchical stochastic structure and are increasingly applied to forest growth and yield modeling [19–23]. Use of *LME* models allows the models to be calibrated by predicting random components from plot-level covariates when a new subject is available and is not used in the fitting of the model by using the empirical best linear unbiased predictors (EBLUPs) [22; 24–26].

The main purpose of this research was to develop an individual tree crown width model for *C*. *lanceolata* in Fujian province, southeast China, on the basis of data derived from 55 sample plots. A one-level (plots effects) linear mixed modeling approach was applied to the hierarchical structure of the data. This diminished the level of variance among the sampling units. Our preliminary analysis showed that the *LME* model effectively removed the heteroscedasticity and spatial correlation in the data and therefore could be an important tool for the sustainable management of China-fir within the study area. The predictive ability of the developed model and the applicability of the *LME* model were demonstrated using separate validation data.

## Materials and Methods

### Data

The pure China-fir stands are located in Jiangle County (117°05′-117°40′E, 26°26′-27°04′N), Fujian Province, southeast China. The soil type is red soil, the average annual precipitation is approximately 1699 mm, the annual mean frost-free season is 287 days, and the annual mean temperature is 18.7°C.

**Fig 1:**
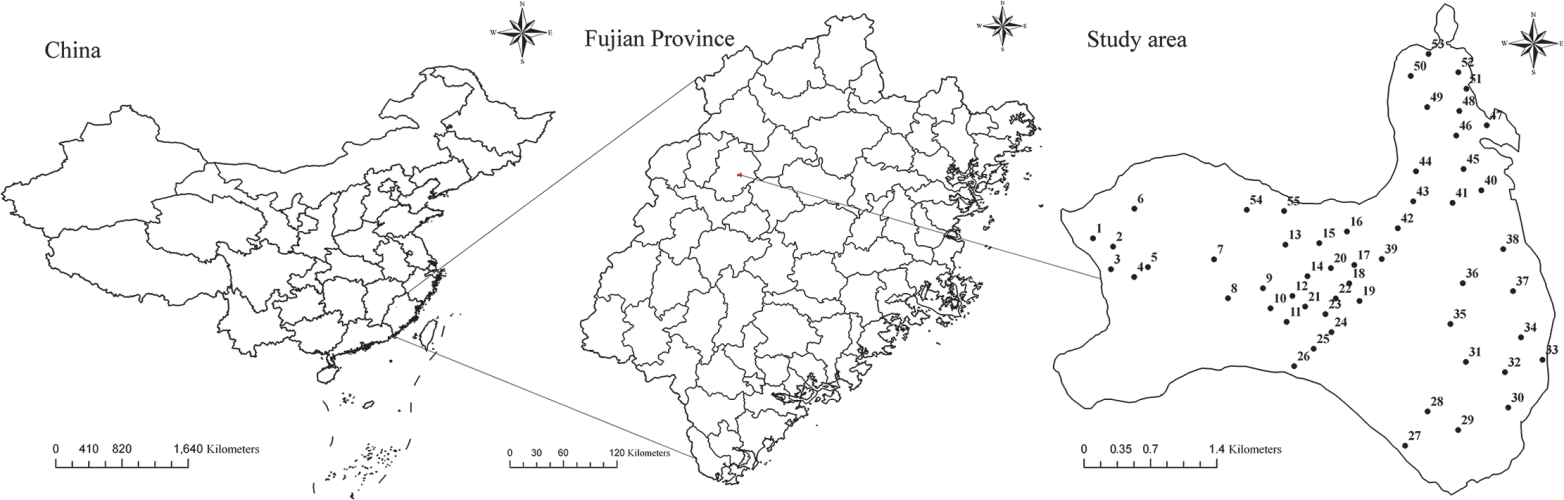
Fifty-five sample plots of pure China-fir plantation stands.

**Fig 2:**
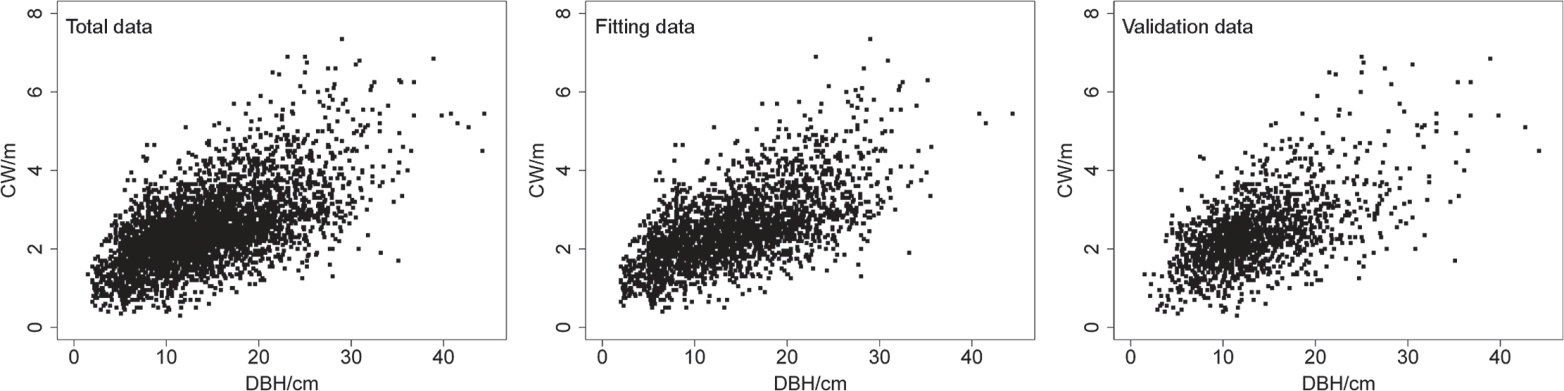
Plots of crown width against DBH for China-fir.

Data from four thousand one hundred ninety-nine trees were obtained from 55 single-species plots of plantation-grown China-fir on the Jiangle state-owned forest farm in Fujian Province, southeast China (Fig 1). The Jiangle state-owned forest farm issued permission for each location, and the field studies did not involve endangered or protected species. The sample plots were square and varied in size from 400 to 600 m^2^. All standing live trees (height > 1.3 m) on the plots were measured for DBH (outside bark), tree height, height to crown base (height above ground to crown base) and crown width. Three to five dominant trees on each plot were chosen to calculate plot dominant height and diameter. Crown width was taken as the arithmetic mean of two crown widths, obtained from measurements of four crown radii in four directions (from the east, west, south and north to the center of tree, respectively) representing two perpendicular azimuths [8]. The crown width data were randomly divided into two groups; 75% of the points were used for model fitting, and 25% were used for model validation, which can be claimed as independent. The fitting and validation data consisted of 2587 trees from 39 plots and 1613 trees from 16 plots, respectively. Summary statistics for both the fitting and validation data are shown in Table 1. The crown data are graphically depicted in Fig 2.

**Table 1:** Summary statistics for increments datasets.

Variables	Fitting data				Validation data			
	Mean	Min	Max	sd	Mean	Min	Max	sd
**CW (m)**	2.53	0.4	7.4	0.95	2.42	0.3	8.2	0.93
**DBH (cm)**	14.44	2.0	44.4	6.75	13.54	1.5	44.2	5.84
***H* (m)**	13.36	1.2	36.5	5.56	12.11	1.3	30.8	5.35
**HCB (m)**	7.68	0.1	21.5	4.08	6.98	0.3	19.8	4.52
**DH (m)**	18.01	6.9	30.3	4.93	15.78	6.5	26.2	5.78
**DD (cm)**	21.43	8.0	38.6	5.13	20.43	10.1	36.0	6.88
***A* (yr)**	22.63	7.0	49.0	9.43	18.10	5.0	40.0	8.73
**SI (m at 20 years)**	17.13	12.0	24.0	3.69	16.89	12.0	22.0	2.86
**SD (trees ha** ^**-1**^ **)**	2311	617	4500	1044.85	2862	467	4400	907.91
**QMD (cm)**	15.03	4.8	25.2	4.64	13.80	9.9	26.9	3.50
**MH (m)**	14.01	4.2	21.3	4.00	12.06	5.0	21.4	4.25
**BA (m** ^**2**^ **·ha** ^**-1**^ **).**	29.54	3.4	68.0	16.11	42.4	16.9	99.82	20.11

### Methods

#### The covariate selection

DBH is an important tree characteristic and the variable that has the greatest correlation with crown width [27]. In addition to DBH, CW is explained by other tree and stand attributes, [9; 28] such as a reduction in growth from increases in stand density, SD (tree ha^-1^) and basal area, BA (m^2^·ha^-1^) [22; 29]. In addition, CW is also influence by tree size variables, such as sample tree height (H) and height to crown base (HCB), and stand variables, such as stand age (A), plot dominant height, DH (m), plot dominant diameter at breast height, DD (cm), plot quadratic mean diameter, QMD (cm) [28; 30; 31], plot mean height, MH (m), and site index, SI (m at 20 yr).

#### Crown width multiple linear model

Independent variables were identified and a backward stepwise linear regression routine that started with all candidate variables, tested the deletion of each variable using a chosen model comparison criterion, deleted the variable (if any) whose removal improved the model the most, and repeated this process until no further improvement was possible, was applied to reduce the number of chosen variables to avoid overfitting. Variance inflation factors (VIF<10), which provide an index that measures how much the variance of an estimated regression coefficient is increased because of collinearity, were also computed to reduce the number of chosen variables to avoid multicollinearity, which could result in numerically unstable estimates of the regression coefficients. Stepwise regression fits an observed dependent dataset using a linear combination of independent variables. The statistical methods were implemented in R, which is a free software environment for statistical computing and graphics [32]. The dependent variable is determined from a linear equation combining the values of the independent dataset with coefficients established by the regression. The statistical results were assessed in terms of the absolute mean residual (*AMR*), root mean square error (*RMSE*), and the adjusted coefficient of determination (adj-*R* ^2^), which accounts for the number of predictors. The calculation formulas of these statistics are listed as follows:

$$AMR=\sum_{i=1}^{M}\sum_{j=1}^{n_{i}}\frac{\left|y_{ij}-{\hat{y}}_{ij}\right|}{n_{i}}$$ 1

$$RMSE=\sqrt{\sum_{i=1}^{M}\sum_{j=1}^{n_{i}}\frac{{\left(y_{ij}-{\hat{y}}_{ij}\right)}^{2}}{n_{i}-r}}$$ 2

$$\mathrm{adj-}R^{\text{2}}=1-\left(n_{ij}-1\right)\left[\frac{\sum_{i=1}^{M}\sum_{j=1}^{n_{i}}\frac{{\left(y_{ij}-{\hat{y}}_{ij}\right)}^{2}}{n_{i}-r}}{\sum_{i=1}^{M}\sum_{j=1}^{n_{i}}{\left(y_{ij}-\bar{y}\right)}^{2}}\right]$$ 3

where *M* is the number of plots, *n*_*i*_ is the number of observations in plot *i*, *r* is the number of parameters in the model, *y*_*ij*_ is the crown width of the *j*th tree taken from the *i*th plot, *ŷ*_*ij*_ is the crown width prediction, and $\bar{y}$ is the average of observations. The accuracy of the models was tested against the fitting data and against independent validation data from the same plot [23].

#### *LME* model method

Available data were from measurements of trees located in sample plots. Because of this nested structure, there is high correlation among observations taken from the same plot. To alleviate this issue, a linear mixed-effects model approach has been proposed by other authors [10; 33]. For a single level of grouping, a general expression for a *LME* model can be defined as [17; 20; 34]:

$$\begin{array}{c} {\text{CW}}_{ij}=X_{ij}\beta +Z_{ij}b_{ij}+\varepsilon_{ij},\;i=1,...,M\;,\;j=1,...,n_{i}\\ b_{ij}∼N\left(0,D\right),\;\varepsilon_{ij}∼N\left(0,R_{ij}\right) \end{array}$$ 4

where CW_*ij*_ is the crown width of the *j*th tree taken from the *i*th plot, *β* is the *p*-dimensional vector of fixed effects (where *p* is the number of fixed-effects parameters in the model), *b*_*ij*_ is the *q*-dimensional vector of random effects associated with plot *i* that is assumed to follow a normal distribution with mean zero and a variance-covariance matrix *D* (where *q* is the number of random-effects parameters in the model), *X*_*ij*_ (of size *n*_*i*_×*p*) and *Z*_*i*_ (of size *n*_*i*_×*q*) are known fixed-effects and random-effects regressor matrices, and *ε*_*ij*_ is the *n*_*i*_-dimensional within-group error vector with a spherical Gaussian distribution [35], which is assumed to be normally distributed with zero expectation and a positive-definite variance-covariance structure *R*_*ij*_, generally is a *n*_*i*_ × 1 vector for the residual items [*e*_*i*1_, *e*_*i*2_, *e*_*i*3_,…, *e*_*i*j_,…, $e_{in_{i}}$]^T^ [36]. Both the random-effects *b*_*ij*_ and the within-group errors *ε*_*ij*_ are assumed to be independent for different groups and to be independent of each other for the same group.

#### Ascertainment of mixed parameters

To fit the mixed-effects models, the key question is which parameters in the model should be considered as random effects and which ones could be treated as purely fixed effects. Generally, an alternative model-building approach is to start with a model with random effects for all parameters and then examine the fitted object to decide which, if any, of the random effects can be eliminated from the model [18]. Therefore, different combinations of model parameters were tested to ascertain their importance with respect to crown width, and the best model was selected by Akaike’s information criterion (AIC) [37], Bayesian information criterion (BIC) [38] and -2 logarithm likelihood (-2 LL) [31]. The less criteria a model has, the better it performs. An appropriate variance function structure for *LME* models were determined by a likelihood ratio test (LRT) [18; 39]. All *LME* models presented in this paper were fitted using the *LME* function in the R statistical software environment.

#### Determining the structure of *R*

This special matrix structure ***R*** (which is allowed to depend on both random and fixed effects, as well as on a set of common but unknown parameters) can include both correlation effects and weighting factors to account for within-group heteroscedasticity and spatial correlation [35; 36; 40]. A general expression for the matrix is given by [40; 41]:

$$R=\sigma^{2}G^{0.5}IG^{0.5}$$ 5

where (in this case) for tree *j* in plot *i*, with *n*_*i*_ increment, **R** is the *n*_*i*_×*n*_*i*_ intraindividual variance- covariance matrix which defines within-group variability, **G** is a *n*_*i*_ ×*n*_*i*_ diagonal matrix of the within-group error variance structure (heteroscedasticity), **I** is a *n*_*i*_×*n*_*i*_ matrix showing the within-group autocorrelation structure of error, and σ^2^ is a scaling factor for the error dispersion [10]. To remove variance heterogeneity, we used the power function, exponential function and constant plus power function as the variance functions to fit crown width models [18].

$$V\left(\varepsilon_{ij}\right)=\sigma^{2}{\text{DBH}}_{ij}^{2\delta }$$ 6

$$V\left(\varepsilon_{ij}\right)=\sigma^{2}\;\text{exp}\left(2\delta {\text{DBH}}_{ij}\right)$$ 7

$$V\left(\varepsilon_{ij}\right)=\sigma^{2}\;{\left(\delta_{1}+{\text{DBH}}_{ij}^{\delta_{2}}\right)}^{2}$$ 8

Correlation structures were used to address the within-tree spatial correlations observed in the data [42; 43]. A method was selected from among three commonly used approaches: the first-order autoregressive structure [AR(1)], a combination of first-order autoregressive and moving average structures [ARMA(1,1)], and the compound symmetry structure (CS) [18].

$$AR\left(1\right)=\sigma^{2}\left[\begin{array}{ccc} 1 & \rho & \rho^{2}\\ \rho & 1 & \rho \\ \rho^{2} & \rho & 1 \end{array}\right]$$ 9

$$ARMA\left(1,\;1\right)=\sigma^{2}\left[\begin{array}{ccc} 1 & \gamma & \gamma \rho \\ \gamma & 1 & \gamma \\ \gamma \rho & \gamma & 1 \end{array}\right]$$ 10

$$CS=\left[\begin{array}{ccc} \sigma^{2}+\sigma_{1} & \sigma_{1} & \sigma_{1}\\ \sigma_{1} & \sigma^{2}+\sigma_{1} & \sigma_{1}\\ \sigma_{1} & \sigma_{1} & \sigma^{2}+\sigma_{1} \end{array}\right]$$ 11

where *ρ* is the autoregressive parameter, *γ* is a moving average component, and σ_1_ is the residual covariance [44; 45].

#### Parameter estimation

The parameters in the equations were estimated by maximum likelihood (ML) using the Lindstrom and Bates (LB) algorithm implemented in the R *LME* function [17; 18]. The LB algorithm and *LME* function are detailed in several articles; see, for example, [17; 18].

A key question in fitting the *LME* models is to estimate the random effects parameters. In this study, they can be calculated with the information from measured trees, such as the measurements of CW and DBH, by the Empirical Best Linear Unbiased Predictors (EBLUPs) [34].

$${\hat{b}}_{ijk}\approx \hat{D}{\hat{Z}}^{\text{T}}{\left(\hat{R}+\hat{Z}\hat{D}{\hat{Z}}^{\text{T}}\right)}^{-1}{\hat{\varepsilon }}_{ijk}$$ 12

where $\hat{D}$ is the estimated variance-covariance matrix for the random-effects ${\hat{b}}_{ijk}$, $\hat{R}$ is the estimated variance-covariance matrix for the error term, and $\hat{Z}$ is the estimated partial derivatives matrix with respect to random effects parameters.

## Results

### Selection of the basic crown width model

The following formula is the composition of individual tree size variables and stand variables for predicting crown width using *OLS*:

$$\begin{array}{l} {\text{CW}}_{ij}=\beta_{0}+\beta_{1}{\text{DBH}}_{ij}+\beta_{2}H_{ij}+\beta_{3}{\text{HCB}}_{ij}+\beta_{4}{\text{DH}}_{i}+\beta_{5}{\text{DD}}_{i}+\beta_{6}A_{i}\\ \;+\beta_{7}{\text{SI}}_{i}+\beta_{8}{\text{SD}}_{i}+\beta_{9}{\text{QMD}}_{i}+\beta_{10}{\text{MH}}_{i}+\beta_{11}{\text{BA}}_{i}+\varepsilon_{ij} \end{array}$$ 13

where *β*_0_ - *β*_11_ are the formal parameters.

To avoid overfitting and multicollinearity between independent variables, the backward stepwise linear regression routine and the variance inflation factor were used to reduce the number of chosen variables. In addition, we took into account the biologically reasonable and the factors that exhibited significance (Pr value<0.05) between independent variables. The variable selection process involves a series of steps beginning with the stepwise regression method together with VIF control to identify those variables that may be useful in the model. DH, *A* and MH were removed from Eq 13 because their VIF>10 (VIF_**DH**_ = 27.48, VIF_*A*_ = 13.61, VIF_MH_ = 17.72). As a result, the final diameter growth model for fir plantations can be expressed as:

$${\text{CW}}_{ij}=\beta_{0}+\beta_{1}{\text{DBH}}_{ij}+\beta_{2}H_{ij}+\beta_{3}{\text{HCB}}_{ij}+\beta_{4}{\text{DD}}_{i}+\beta_{5}{\text{SI}}_{i}+\beta_{6}{\text{SD}}_{i}+\beta_{7}{\text{QMD}}_{i}+\beta_{8}{\text{BA}}_{i}+\varepsilon_{ij}$$ 14

The statistics used for the selection of the basic model are shown with equations in Table 2.

**Table 2:** Comparison of fitting statistics and estimated variance components of the models with different alternatives of covariates inclusion, residual variance function and variance components estimation method.

Model	Intercept	DBH	*H*	HCB	DD	SI	SD	QMD	BA
**Eq 14**	1.3450	0.1235	-0.0212	-0.0275	0.0236	-0.0077	2.31×10–5	-0.0127	-0.0105
**(standard error)**	(0.0904***)	(0.0032***)	(0.0050***)	(0.0045***)	(0.0035***)	(0.0037*)	(4.4×10–5*)	(0.0054**)	(0.0009***)
**Eq 14.4**	1.1812	0.1103	0.0073	-0.0238	0.0152	-0.0115	9.07×10–5	-0.0182	-0.0060
**(standard error)**	(0.5102)	(0.0066**)	(0.0080*)	(0.0079)	(0.0193)	(0.0197)	(7.68×10–5**)	(0.0265)	(0.0048)
**Eq 14.4.8**	0.6693	0.1090	0.0085	-0.0217	0.0231	-0.0134	0.0002	-6.93×10–3	-0.0075
**(standard error)**	(0.4490*)	(0.0061***)	(0.0060*)	(0.0062**)	(0.0154*)	(0.0163*)	(0.0001**)	(0.0217*)	(0.0038**)

“*”means Pr value < 0.05

“**” means Pr value < 0.01

“***” means Pr values < 0.001.

### Construction of *LME* models

There would be ninety different combinations of no more than four random-effects parameters for Eq 14 while simultaneously considering plots effects. The *LME* models with more than four random-effects parameters could not reach convergence.

LRT, AIC, BIC and -2 LL statistics were compared between the *LME* models with the best different combinations of random-effects parameters and are shown in Table 3. The model of Eq 14, incorporating plots effects on *β*_0_, *β*_1_, *β*_2_ and *β*_3_ (Eq 14.4), yielded the smallest AIC, BIC and -2 LL and had significant differences when compared to the other *LME* models (Eqs 14.1–14.3) (Pr<0.0001).

$$\begin{array}{l} {\text{CW}}_{ij}=\left(\beta_{0}+u_{0i}\right)+\beta_{1}{\text{DBH}}_{ij}+\beta_{2}H_{ij}+\beta_{3}{\text{HCB}}_{ij}\\ \;+\beta_{4}{\text{DD}}_{i}+\beta_{5}{\text{SI}}_{i}+\beta_{6}{\text{SD}}_{i}+\beta_{7}{\text{QMD}}_{i}+\beta_{8}{\text{BA}}_{i}+\varepsilon_{ij} \end{array}$$ 14.1

$$\begin{array}{l} {\text{CW}}_{ij}=\left(\beta_{0}+u_{0i}\right)+\left(\beta_{1}+u_{1i}\right){\text{DBH}}_{ij}+\beta_{2}H_{ij}+\beta_{3}{\text{HCB}}_{ij}\\ \;+\beta_{4}{\text{DD}}_{i}+\beta_{5}{\text{SI}}_{i}+\beta_{6}{\text{SD}}_{i}+\beta_{7}{\text{QMD}}_{i}+\beta_{8}{\text{BA}}_{i}+\varepsilon_{ij} \end{array}$$ 14.2

$$\begin{array}{l} {\text{CW}}_{ij}=\left(\beta_{0}+u_{0i}\right)+\left(\beta_{1}+u_{1i}\right){\text{DBH}}_{ij}+\left(\beta_{2}+u_{2i}\right)H_{ij}+\beta_{3}{\text{HCB}}_{ij}\\ \;+\beta_{4}{\text{DD}}_{i}+\beta_{5}{\text{SI}}_{i}+\beta_{6}{\text{SD}}_{i}+\beta_{7}{\text{QMD}}_{i}+\beta_{8}{\text{BA}}_{i}+\varepsilon_{ij} \end{array}$$ 14.3

$$\begin{array}{l} {\text{CW}}_{ij}=\left(\beta_{0}+u_{0i}\right)+\left(\beta_{1}+u_{1i}\right){\text{DBH}}_{ij}+\left(\beta_{2}+u_{2i}\right)H_{ij}+\left(\beta_{3}+u_{3i}\right){\text{HCB}}_{ij}\\ \;+\beta_{4}{\text{DD}}_{i}+\beta_{5}{\text{SI}}_{i}+\beta_{6}{\text{SD}}_{i}+\beta_{7}{\text{QMD}}_{i}+\beta_{8}{\text{BA}}_{i}+\varepsilon_{ij} \end{array}$$ 14.4

where *β*_0_ - *β*_8_ are the fixed effects parameters and *u*_0*i*_, *u*_1*i*_, *u*_2*i*_ and *u*_3*i*_ are the random-effects parameters generated by plots effects on *β*_0_, *β*_1_, *β*_2_ and *β*_3_, respectively.

**Table 3:** Performance criteria of *LME* models for combinations of random effects.

Equation	Mixed parameters	Number of parameters	AIC	BIC	-2 LL	LRT	Pr values
**14.1**	*β*_0_	10	7910.90	7980.64	7888.90	—	—
**14.2**	*β*_0_, *β*_1_	11	7820.25	7902.68	7794.25	94.64	<0.0001
**14.3**	*β*_0_, *β*_1_, *β*_2_	12	7751.38	7852.83	7719.38	76.26	<0.0001
**14.4**	*β*_0_, *β*_1_, *β*_2_ *β*_3_	13	7723.20	7850.01	7683.20	34.80	<0.0001

### *LME* model with heteroscedasticity and spatial correlation

We used the power function, the exponential function or the constant plus power function as the variance functions and AR, ARMA(1,1) or CS as the correlation structure to update Eq 14.4 to reduce heteroscedasticity and spatial correlation. The *LME* models with variance functions and correlation structures are shown in Table 4. In this study, Equation 14.4.1 is the same as Eq 14. The best models were chosen with the smallest AIC, BIC and -2 LL. Thus, the final models of plots effects are:

Equation14.4+Equation6 14.4.2

Equation14.4+Equation7 14.4.3

Equation14.4+Equation8 14.4.4

Equation14.4+Equation9 14.4.5

Equation14.4+Equation10 14.4.6

Equation14.4+Equation11 14.4.7

Equation14.4+Equation6+Equation10 14.4.8

**Table 4:** Comparisons of intercept effect mixed model performance for fir plantations diameter increment data with different within-tree correlation structures and different variance functions.

Equation	Variance function	Correlation structure	Number of parameters	AIC	BIC	-2LL	LRT	Pr values
**14.4.1**	Homogeneous	Independent	13	7723.20	7850.01	7683.20	—	—
**14.4.2**	Power	Independent	14	7458.74	7591.89	7416.74	266.46 ^a^	<0.001
**14.4.3**	Exponent	Independent	14	7422.96	7556.11	7380.96	302.24 ^a^	<0.001
—	—	—	—	—	—	—	436.97 ^b^	<0.001
**14.4.4**	Const plus power	Independent	15	7444.24	7573.73	7400.24	322.95 ^a^	<0.001
**14.4.5**	Homogeneous	AR(1)	14	7473.63	7606.78	7431.63	251.56 ^a^	<0.001
—	—	—	—	—	—	—	368.08 ^b^	<0.001
**14.4.6**	Homogeneous	ARMA(1,1)	14	7356.06	7495.55	7312.06	371.13 ^a^	<0.001
**14.4.7**	Homogeneous	CS	14	7725.20	7858.35	7683.20	3.31×10^–6^ ^a^	0.9985
**14.4.8**	Exponent	ARMA(1,1)	16	6989.99	7135.82	6943.99	739.21 ^a^	<0.001

^a^ Likelihood ratio is calculated with respect to Equation 14.4.1

^b^ Likelihood ratio is calculated with respect to Eq 14.4.8

### Parameter estimates

The *LME* CW model with plots effects is then defined by the following expression:

$$\begin{array}{l} {\text{CW}}_{ij}=\left(0.8247+u_{0i}\right)+\left(0.1093+u_{1i}\right){\text{DBH}}_{ij}+\left(0.0095+u_{2i}\right)H_{ij}+\left(-0.0219+u_{3i}\right){\text{HCB}}_{ij}\\ \;+0.0181{\text{DD}}_{i}-0.0105{\text{SI}}_{i}+0.0004{\text{SD}}_{i}-0.0121{\text{QMD}}_{i}-0.0064{\text{BA}}_{i}+\varepsilon_{ij} \end{array}$$ 15

Where

$$\begin{array}{c} u_{i}=\left[\begin{array}{c} u_{0i}\\ u_{1i}\\ u_{2i}\\ u_{3i} \end{array}\right]∼\text{N}\left\{\left[\begin{array}{c} 0\\ 0\\ 0\\ 0 \end{array}\right],D=\left(\begin{array}{cccc} 0.5323 & 0.0916 & -0.5342 & -0.7542\\ 0.0916 & 0.0360 & -0.5718 & -0.3328\\ -0.5342 & -0.5718 & 0.0262 & 0.3987\\ -0.7542 & -0.3328 & 0.3987 & 0.0323 \end{array}\right)\right\}\\ \varepsilon_{ij}∼N\left(0,R_{ij}=0.3592G_{i}^{0.5}I_{i}G_{i}^{0.5}\right)\\ V\left(\varepsilon_{ij}\right)=\text{exp}\left(\text{0}{\text{.4366DBH}}_{ij}\right)\\ ARMA\left(1,\;1\right)=\left[\begin{array}{ccc} 1 & 0.9610 & -0.7731\\ 0.9610 & 1 & 0.9610\\ -0.7731 & 0.9610 & 1 \end{array}\right] \end{array}$$

### Model prediction

The predictive ability of Eq 14 was evaluated using prediction procedures and Eq 1–3 on both fitting and validation data. The performance of the *LME* models, with and without modeling the error structure, was evaluated using cross-validation procedures for both fitting and validation data; the random effects were predicted with the EBLUPs (Eq 12), using the measurement data.

**Fig 3:**
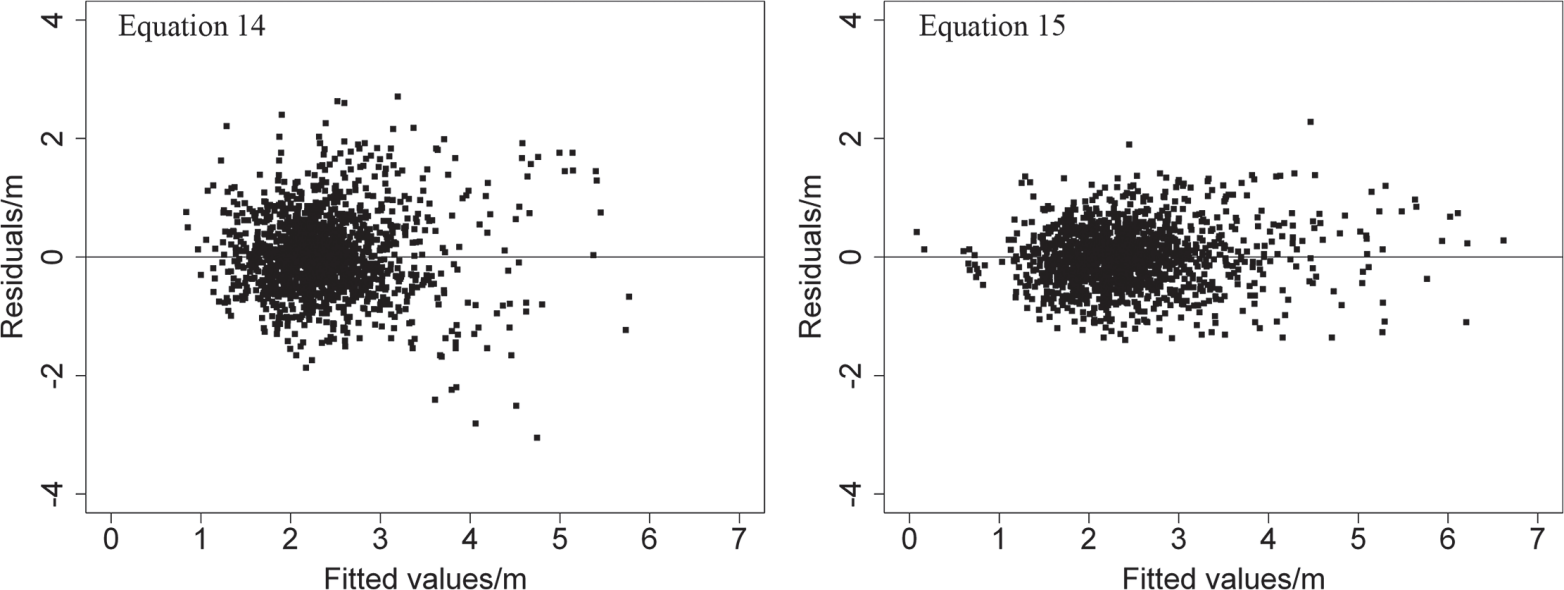
Distribution of residuals for two equations fitting crown width of China-fir trees.

**Fig 4:**
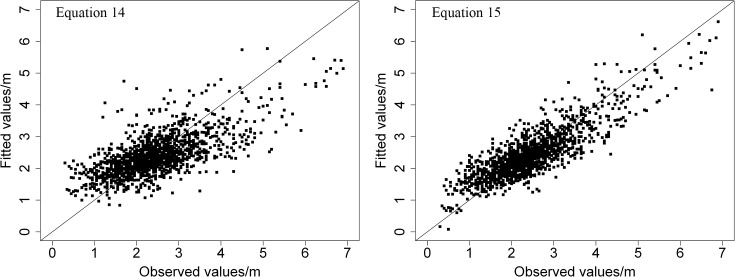
Fitted values of two equations for crown width of China-fir trees against observed values.

Table 5 lists the three prediction statistics of Eq 14, Eq 15 and Eq 15 without random effects for both fitting and validation data. Compared with Eq 14, Eq 15 had a higher adj-*R* ^2^, 0.7226 compared to 0.4733, and lower *RMSE*, 0.4854 compared to 0.6688, and *AMR*, 0.3688 compared 0.4954, for the validation data. In Fig 3, the residuals of Eq 14 and 15 are plotted against the fitted values. The fitted values of these equations are plotted against the observed values in Fig 4. Based on the above analysis, we can conclude that Eq 15, incorporating the random effects plots, was better than Eq 14. The *LME* model provides a model for predicting the expected values of crown width for individual trees of China-fir in the single-species plantations of the study area.

**Table 5:** Evaluation indices of each model.

Model	Effects	Fitting data			Validation data		
		*AMR*	*RMSE*	adj-*R* ^2^	*AMR*	*RMSE*	adj-*R* ^2^
**Eq 14**		0.5306	0.6914	0.4694	0.4954	0.6688	0.4733
**Eq 15**	**Mixed effects**	0.4027	0.5070	0.7147	0.3688	0.4854	0.7226

## Discussion

In this study, a backward stepwise linear regression was used to establish a multiple linear individual tree crown width model for China-fir. The relative importance of explanatory variables used to predict the crown width were assessed. Generally, DBH is the tree size variable most related to crown width [27]. In addition to DBH, the tree size variables (such as H and HCB in this study) and stand variables (such as DH in this study) are also obvious factors affecting the crown width [20; 22; 46; 47]. Both stand and tree development are linked to the DH because it is a measureable stand characteristic that indicates site quality in terms of the stand growth and yield capacity [20]. The variables H and HCB showed a significant effect on the crown width because they are closely related to tree size and have an important role in crown fire initiation and spread [47; 48]. Therefore, we selected the diameter at breast height, tree height, height to crown base, plot dominant height, plot dominant diameter at breast height, stand age, site index, stand density, plot quadratic mean diameter, plot mean height and basal area as the independent variables to establish an individual tree crown width model. However, variance inflation factors were used to avoid potential overfitting and multicollinearity.

## Conclusions

Eleven variables were selected in this study to describe crown width (Eq 13) of China-fir in pure plantation stands in Fujian province, southeast China. Then, the backward stepwise linear regression routine and the variance inflation factor were used to reduce the number of chosen variables (Eq 14). The one-level (plot) *LME* model using the variance function structure and correlation structure approach were used to estimate the relationship of the chosen variables with crown width for individual trees. The results showed that the one-level *LME* models with mixed effects, considering variance function structure and correlation structure (Eq 15), provided better model fitting and more precise estimations than the *LME* models without mixed effects (Eq 14) (Table 5 and Figs 3 and 4). Therefore, we recommend using a linear mixed effects modeling approach to build an individual tree crown width model.
